# Heat Treatment of High-Performance Ferritic (HiperFer) Steels

**DOI:** 10.3390/ma16093500

**Published:** 2023-05-01

**Authors:** Bernd Kuhn, Michal Talik

**Affiliations:** Forschungszentrum Jülich GmbH, Institute of Energy and Climate Research (IEK), Microstructure and Properties of Materials (IEK-2), 52425 Juelich, Germany

**Keywords:** HiperFer steel, laves phase strengthening, heat treatment, microstructure, creep

## Abstract

High-performance Ferritic (HiperFer) steels are a novel class of heat-resistant, fully ferritic, Laves phase precipitation hardened materials. In comparison to conventional creep strength-enhanced 9–12 wt.% Cr ferritic–martensitic steels, HiperFer features increased mechanical strength, based on a thermodynamically stable distribution of small (Fe,Cr,Si)_2_(Nb,W) Laves phase precipitates, and—owing to its increased chromium content of 17 wt.%—improved resistance to steam oxidation, resulting in superior temperature capability up to 650 °C. Previous publications focused on alloying, thermomechanical processing, and basic mechanical property evaluation. The current paper concentrates on the effect of heat treatment on microstructural features, especially Laves phase population, and the resulting creep performance. At 650 °C and a creep stress of 100 MPa, an increase in rupture time of about 100% was achieved in comparison to the solely thermomechanically processed state.

## 1. Introduction

In the temperature range from 560 to 620 °C, current thermal power conversion equipment mainly features so-called creep strength-enhanced ferritic–martensitic (CSEF) 9–12 wt.% Cr structural steels [1,2]. This material class is mainly strengthened by solid solution, MX (M: Metal, X: C/N), and M_23_C_6_ particle hardening. Due to insufficient resistance to steam oxidation, the 9 Cr steels are typically especially limited to application temperatures of less than 620 °C [2,3]. The 12 wt.% Cr steels provide improved oxidation resistance at temperatures above 620 °C but suffer decreasing long-term creep strength because of Z-phase formation [4]. The Z-phase is a coarse Cr(V, Nb)N and consumes fine, strengthening MX particles [5,6], deterring microstructural stability and therefore creep performance. For these reasons, CSEF steel development stagnated for at least 20 years, necessitating new approaches.

The novel “HiperFer” (High-performance Ferrite) steels offer superior resistance to fatigue [7,8], long crack propagation [8], creep [9,10,11], and steam oxidation [12], provided by its fully ferritic, stainless (17 wt.% Cr) matrix and strengthened by solid solution (by W, Nb, and Cr) and (Fe,Cr,Si)_2_(Nb,W) Laves phase precipitation [8,9,10,11,12]. In the as-rolled, i.e., thermomechanically processed (TMP) state, the creep strength of these grades at least levels out grade 92 steel [8,9], with its steam oxidation resistance being superior to even the 12 wt.% Cr CSEF steels [12]. The implementation of a growing share of intermitting regenerative sources of power into the grid necessitates improved operational flexibility today already. In future, thermal power equipment operational flexibility—and consequently increased resistance to thermomechanical fatigue, and additional resistance to long crack propagation in steam power cycles under superimposed steam oxidation attack [13,14]—will be of the highest importance [15]. Thus, improved fatigue performance was the main goal in the development of the HiperFer grades. Nevertheless, high creep strength remained an important issue because it results in thinner wall sections; thus, it also results in diminished thermomechanical loading of thick section parts and furthermore in lower investment costs because of decreased material consumption.

HiperFer features a fully ferritic matrix (i.e., no γ-iron phase at high temperature), i.e., does not undergo transformation to martensite during cooling down from high temperature. Because of this, it is intrinsically free from so-called Type IV cracking (i.e., the premature creep rupture of weldments [16,17]), because changes in grain morphology of the heat-affected zone do not appear. On the other hand, this characteristic may require special thermomechanical processing (TMP) [9,18] to compensate lacking dislocation density in the early stage of creep deformation. Application of thermomechanically processed materials may be restricted in the production of complexly shaped components or especially when welding is involved (tubing, piping). The current research paper describes suitable heat treatments consisting of recrystallization and subsequent precipitation annealing. While strengthening of HiperFer is dominated by thermomechanically triggered precipitation in the fatigue loading regime, the impact of heat treatment on alloy microstructure becomes most obvious in the achievable creep properties. For this reason, both microstructure and creep response are addressed, and it is demonstrated that TMP is not mandatory for HiperFer steel.

## 2. Materials and Methods

### 2.1. Base Metal Production and Processing

The experimental HiperFer 17Cr2 batch constitutes a comparatively low alloyed variant of this type of steel (cf. [8]). It was produced from high-purity raw materials by VIM (Vacuum Induction Melting) at the Steel Institute (IEHK) of the Northrhine-Westfalian Technical University Aachen (RWTH, Aachen, Germany), Germany (chemical composition given in Table 1; analyzed by Inductively Coupled Plasma Optical Emission Spectroscopy (ICP-OES); C, N levels by infrared absorption).

The ingot was casted to a block dimension of 140 mm × 140 mm × ~525 mm, forged to an 80 mm × 56 mm cross-section slab, and subsequently air-cooled. The slab was then cut into ~135 mm long pieces. Soaking, rolling, and subsequent cooling was performed like summarized in parameter set “_4” in Table 2 (for details on the process, refer to [9]). Rolling resulted in 15 mm thick plate material.

### 2.2. Recrystallization and Precipitation Heat Treatment

Recrystallization (RX) of the plates was achieved at 1075 °C for 15 min, followed by water quenching (WQ). The precipitation heat treatment parameters are given in Table 3.

In order to assess the effect of increased dislocation density from thermomechanical rolling on precipitation kinetics, the precipitation heat treatment trials were also performed on as-rolled (cf. [9] for details) plate material. In HiperFer-type steel, intergranular precipitation of (Fe, Cr, Si)_2_(Nb, W) Laves phase [19,20] particles is faster than intragranular precipitation. Annealing at sufficiently high temperature leads to early formation of Laves phase precipitates at the grain boundaries, which causes depletion of the surrounding matrix in Laves phase-forming elements [21]. This hinders nucleation of intragranular precipitates in close vicinity to the grain boundaries and results in the formation of adjacent precipitation-free zones (PFZs) [18,21]. Plastic deformation at high temperature concentrates in these PFZs [21]. For this reason, PFZ volume has a distinct impact on the creep performance of these Laves phase-strengthened steels [8,19,20,21], and they must be properly controlled by grain size and PFZ width. The “PA stage 1” (540 °C/5 h/WQ, cf. Table 3) was inspired by the heat treatment of Fe_2_Nb Laves phase-strengthened, Fe-rich, carbon-free ferritic steel, where it proved successful in reducing the width of particle free zones along grain boundaries [22]. Furthermore, 540 °C/5 h annealing is effective in reducing both incubation time and mean particle diameter achieved in the following 650 °C annealing step. With 650 °C being the design temperature of HiperFer steel, it was selected as the primary temperature for “PA stage 2” (Table 3) to assess the age-hardening (i.e., on-site or in-process heat treatment during plant commissioning) characteristics of the material. The lower temperature variation was performed to evaluate if the precipitation reactions are still fast enough at 625 °C. Annealing was conducted at 675 °C to rate the impact of increased temperature on precipitate size distribution. The performed variations enable a first estimate of suitable time/temperature combinations for effective heat treatment.

For the investigation of the microstructural state before creep testing (but after heat treatment), annealing specimens (5 × 5 × 5 mm^3^) were prepared from the plate materials and heat treated according to the parameters summarized in Table 3. The 1 h equilibration stage (“Equil. stage” in Table 3) reproduces the holding time performed in the creep experiments (cf. Section 2.3) before applying the creep load and was implemented to ensure the best possible correlation of the initial microstructures and the resulting creep curves.

### 2.3. Mechanical Testing

The specimens for creep testing were machined from the rolled plates (perpendicular to the rolling direction, because in the case of the as-rolled material, the transverse direction gave more conservative results in creep testing) and featured a gauge diameter/length of 6.4/30 mm. Single specimen, lever arm type, constant load creep machines, with continuous measurement of elongation were employed in creep testing. The temperature in the electrical three-zone furnaces was controlled to an accuracy of +/−2 °C by type R (Pt/RhPt) thermocouples, which were attached to the specimen gauge lengths. Heating up to the designated temperature was performed with 5 K/min. A one-hour holding period at 650 °C was implemented for temperature equilibration of the testing set-up before applying the creep load (cf. previous section).

### 2.4. Microstructural Investigation

All the samples for microstructural investigation were mounted in epoxy resin and subsequently ground and polished to a sub-micron finish using colloidal silica suspension for approx. 4 h. After this, the polished specimens were electrolytically etched in 5% H_2_SO_4_ at a voltage of at 1.5 V to increase the particle/matrix contrast. Details on specimen preparation can be found in [20]. A Zeiss Merlin (Oberkochen, Germany) field emission, scanning electron microscope was utilized for observation. High-resolution micrographs were post-processed, binarized, and analyzed by the freely available image analysis software ImageJ (Version 1.53a) [23] concerning precipitate size evolution, following the method outlined in [21]. Grain boundaries (both high-angle and sub-grain) were excluded from the evaluation of particle size distribution.

## 3. Results and Discussion

### 3.1. Impact of Heat Treatment on Initial Microstructure

**Figure 1:**
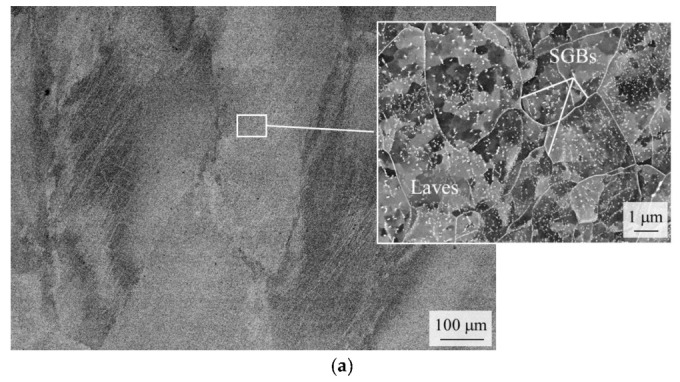

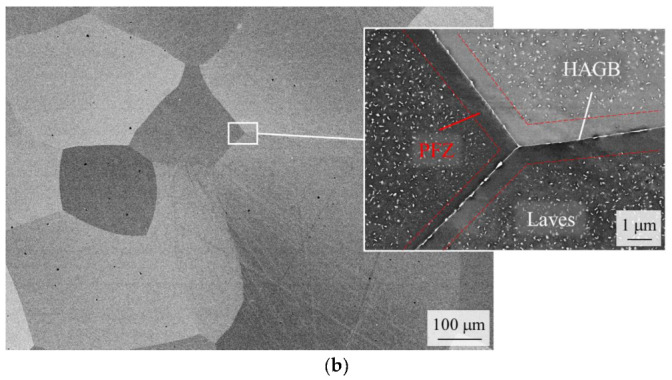
Typical scanning electron micrographs of HiperFer steel after precipitation heat treatment (540 °C/5 h/WQ + 650 °C/1 h/WQ + Equil. stage). (**a**) As-rolled (TMPed) material: Deformed grain structure, detail, Laves phase-covered sub-grain boundaries, inhomogeneous distribution of intragranular Laves phase precipitates. (**b**) Recrystallized material: Globular grain structure, Laves phase-covered high-angle grain boundaries with particle-free zones alongside, homogeneously distributed intragranular Laves phase precipitates.

As already mentioned, HiperFer steel does not present an austenite phase at any temperature and consequently does not transform martensitically (γ-iron => α’-iron) during cooling from processing. For this reason, the achievable mechanical properties either directly depend on thermo-mechanical processing (covered in [8,9]), or can be adjusted by heat treatment, consisting of recrystallization and subsequent precipitation annealing. The heat treatment trials concentrated on achieving fully globular recrystallization of the deformed grain structure, homogenous distribution of small inter- and intragranular Laves phase precipitates, and reduction in the PFZ width alongside high-angle grain boundaries. Figure 1 depicts typical micrographs taken after precipitation heat treatment.

After precipitation annealing at 540 °C for 5 h (WQ) and subsequently at 650 °C for 1 h (WQ), the as-rolled material (Figure 1a) displays deformed grain structure, Laves phase-covered sub-grain boundaries (SGBs), and small—but inhomogeneously distributed—intragranular Laves phase precipitates. The recrystallized (1075 °C/15 min/WQ) material presents globular grain morphology without sub-grains, Laves phase-covered high-angle grain boundaries (HAGBs) with particle-free zones (PFZs) alongside, and a homogenous distribution of intragranular Laves phase precipitates.

### 3.2. Evaluation of the Effect of Heat Treatment on Microstructure

#### 3.2.1. As-Rolled Material

**Figure 2:**
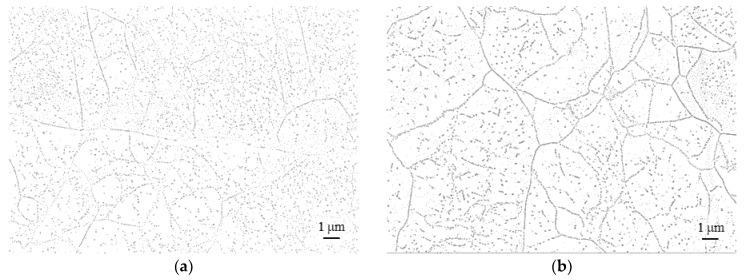

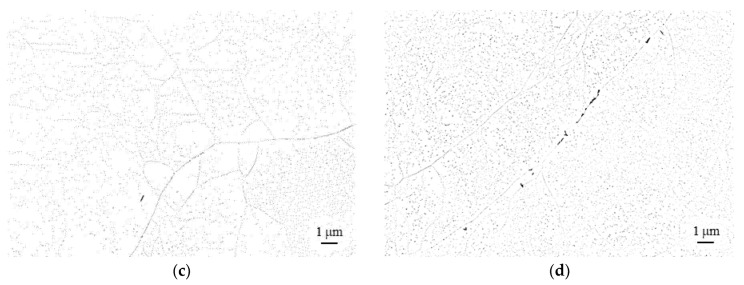
Post-processed, binarized, and inverted (for better particle visibility) high-resolution scanning electron micrographs of Laves phase precipitates in as-rolled HiperFer 17Cr2 steel: (**a**) Equil. Stage; (**b**) 650 °C/1 h/WQ + Equil. Stage; (**c**) 540 °C/5 h/WQ + Equil. Stage; (**d**) 540 °C/5 h/WQ + 650 °C/1 h/WQ + Equil. stage.

Post-processed, binarized, and inverted images of high-resolution scanning electron micrographs of rolled and precipitation-annealed material are depicted in Figure 2. After “single” (Figure 2a: “Equil. Stage” only, i.e., 650 °C/1 h/WQ) and “double” (Figure 2b: 650 °C/1 h/WQ + “Equil. Stage”; i.e., effectively: 2 h at 650 °C/WQ) annealing at 650 °C, the sub-grain boundaries were populated by Laves phase particles and an inhomogeneous distribution of intragranular precipitates became obvious. 

**Figure 3:**
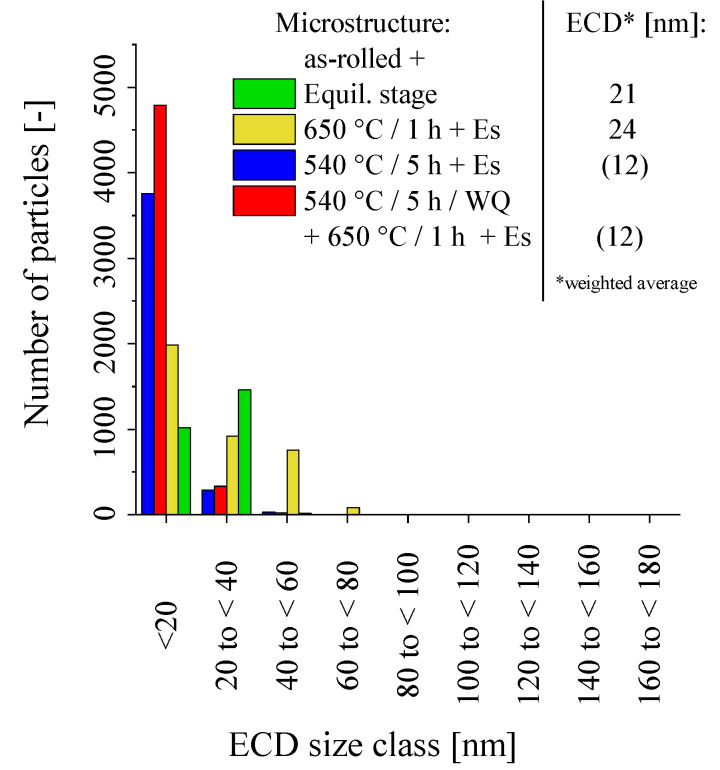
Particle size distributions in rolled (TMPed) material after various precipitation heat treatments (* ECD: Equivalent circle diameter, i.e., diameter of a circular particle of equivalent area).

Additional pre-annealing at 540 °C led to particle refinement (Figure 2c,d). While the weighted average particle sizes were 21 (Figure 2a: 1 h at 650 °C) and 24 nm (Figure 2b: 2 h at 650 °C) after 650 °C annealing, the mean particle size dropped below 20 nm (it must be noted that such small particles cannot be analyzed reliably from SEM micrographs. The results indicated for “540 °C/5 h + Es” and “540 °C/5 h + 650 °C/1 h + Es” in Figure 3 by trend do make sense and are correspondable to the creep response encountered. Nevertheless, for these two heat treatment conditions, neither the absolute values given in the size distributions nor for the mean particle diameters should be taken literally) with the implementation of the 540 °C pre-annealing step (Figure 2c,d and Figure 3). The particle-refining effect of low-temperature pre-annealing furthermore diminished the visibility of grain and sub-grain boundaries.

The size distributions in Figure 3 clearly support the particle refining effect of 540 °C pre-annealing. The 540 °C/5 h/WQ treatment includes the multitude of precipitates ranges at sizes below 20 nm, while the larger sizes are underrepresented. Without the pre-annealing step, the size distributions shift towards lower numbers of particles below 20 nm in favor of the larger sizes. Prolonged time at 650 °C increases the absolute number of precipitates (cf. Figure 3: “Equil. stage” and “650 °C/1 h/WQ + Equil. stage”). After two hours effectively at 650 °C (i.e., 650 °C/1 h/WQ + Equil. stage), the numbers of precipitates smaller than 20 nm—as well as in the size ranges from 20 to 40 nm and from 40 to 60 nm—increase simultaneously, which indicates that the precipitation process was still not finished.

#### 3.2.2. Recrystallized Material

Mechanical properties, dependent on thermomechanical processing, may restrict the utilization of HiperFer to “non-welding” applications, i.e., where component manufacturing does not cause significant microstructural changes. Furthermore, the complexity and additional cost implied with TMP may prohibit market entry or deeper market penetration. These putative drawbacks can be resolved by recrystallization (RX) and subsequent precipitation annealing (PA) heat treatment. Tailored precipitation kinetics of the Laves phase enable simplified, short-term PA in the envisaged application temperature range.

**Figure 4:**
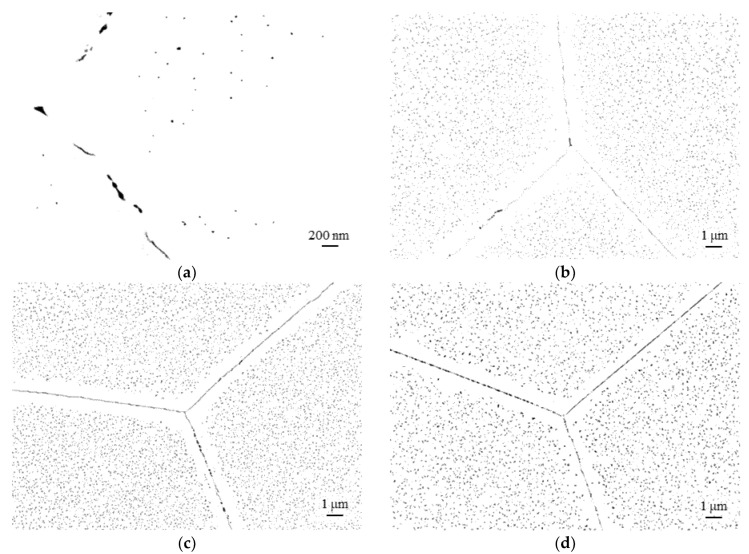

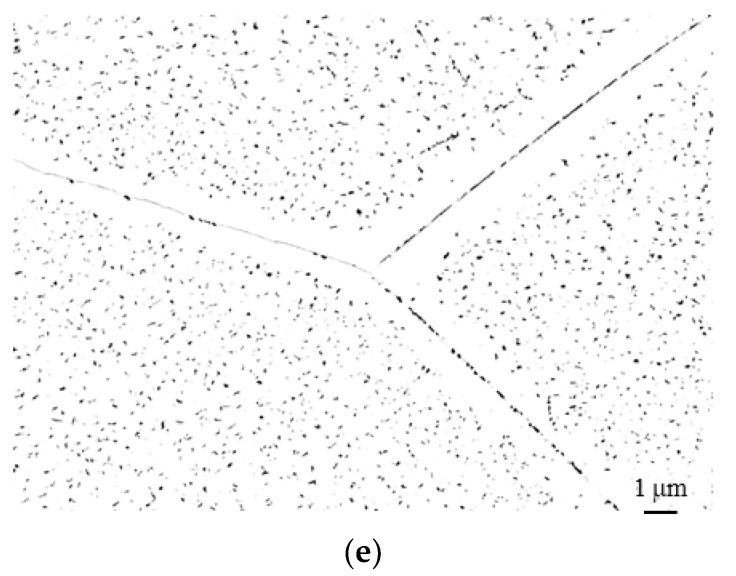
Post-processed, binarized and inverted high-resolution scanning electron micrographs of Laves phase precipitates in recrystallized (1075 °C/15 Min./WQ) HiperFer 17Cr2 steel: (**a**) 540 °C/5 h/WQ + 650 °C/1 h/WQ + Equil. Stage; (**b**) 540 °C/5 h/WQ + 650 °C/2 h/WQ + Equil. Stage; (**c**) 540 °C/5 h/WQ + 625 °C/10 h/WQ + Equil. Stage; (**d**) 540 °C/5 h/WQ + 650 °C/10 h/WQ + Equil. Stage; (**e**) 540 °C/5 h/WQ + 675 °C/10 h/WQ + Equil. stage.

While in the rolled state, intragranular precipitates preferentially nucleate at dislocations [19,20], and the intragranular precipitate population, apart from the occurrence of PFZs at high-angle grain boundaries, generally appears more homogenous (cf. Figure 4) with RX included. RX causes prolonged incubation time. Consequently, the particles are smaller by trend with RX involved (cf. microstructures of corresponding heat treatment schedule: “540 °C/5 h/WQ + 650 °C/1 h/WQ + Es” (Figure 2d) vs. “RX + 540 °C/5 h/WQ + 650 °C/1 h/WQ + Es” (Figure 4a)).

**Figure 5:**
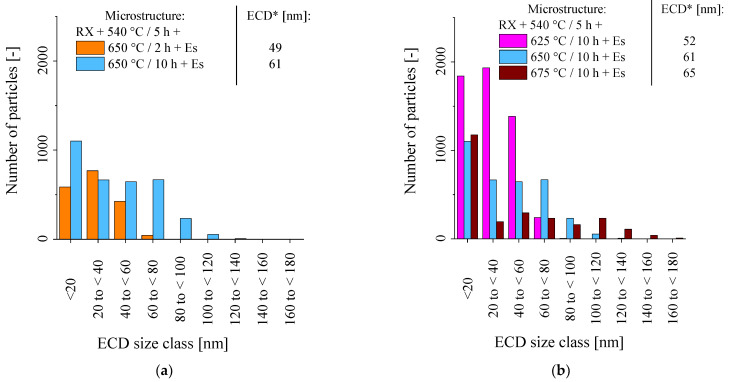
Particle size distributions in recrystallized material after various precipitation heat treatments (*ECD: Equivalent circle diameter, i.e., diameter of a circular particle of equivalent area). (**a**) Impact of holding time in 650 °C annealing; (**b**) Impact of annealing temperature at a fixed holding time of 10 h.

Figure 5a compares the size distributions after prolonged annealing at 650 °C. After 10 h, the number of precipitates increased in all the size ranges. The precipitation process thus obviously continued over the evaluated period.

Figure 5b displays the expected effect of varying annealing temperature at given times. While at 625 °C the majority of precipitates prevails in the size classes below 100 nm, annealing at 650 °C and 675 °C increasingly shifts the precipitates towards the larger size ranges (cf. Figure 4c–e). Interestingly, the number of particles below 20 nm is more or less comparable at 650 and 675 °C, while in the intermediate size ranges (20–100 nm), the number of particles is smaller at 675 °C (Figure 5b). This may indicate that new, small particles still nucleate, while the initial ones are already coarsening. Furthermore, the particles near high-angle boundaries may dissolve faster at 675 °C, which leads to the visibly wider PFZs in Figure 4e and may explain the lower number of particles.

Precipitation heat treatment is effective in increasing both the mechanical properties of as-rolled and recrystallization-annealed HiperFer steel. As a summary, it can be stated that effective precipitation heat treatment is possible in a comparatively wide parameter window concerning temperature (from 625 to 675 °C) and time (from 1 to 10 h). With this in mind, even precipitation heat treatment during plant commissioning would be feasible for processes which feature peak temperatures above 620 °C.

### 3.3. Correlation of Microstructure and Creep Response

#### 3.3.1. As-Rolled Material

**Figure 6:**
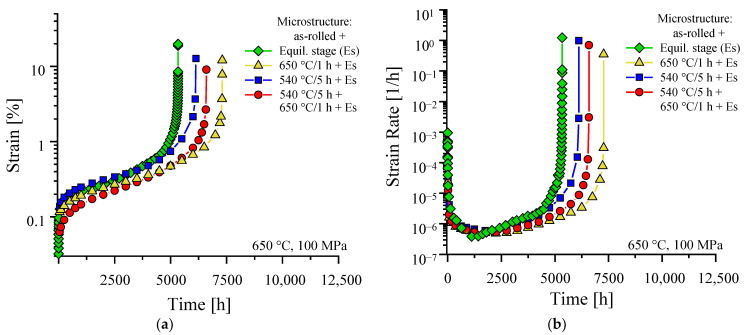
The 650 °C/100 MPa creep (**a**) and creep rate (**b**) curves of as-rolled (TMPed) material after various precipitation heat treatments.

The creep and creep rate curves in Figure 6 do well reflect the initial microstructure conditions after heat treatment. In the particle size histogram (Figure 3), the as-rolled material exhibits the lowest overall number of particles. Nevertheless, the high dislocation density, originating from the rolling process and preserved by subsequent WQ, sufficiently strengthens the as-rolled material (“Es” in Figure 6a). HiperFer steel does not exhibit a classical secondary stage of creep [8,24]. This is caused by thermomechanically triggered precipitation upon loading and during primary creep, which leads to a continuous drop in creep rate (Figure 6b) until the onset of the tertiary stage (appr. 1120 h in case of the as-rolled material). At 650 °C and a testing stress of 100 MPa, the as-rolled material reached a rupture time of 5334 h.

With a single step, low-temperature precipitation heat treatment of 5 h at 540 °C (and subsequent WQ) included, part of the excess dislocations from rolling recovers, the incubation time of precipitation at 650 °C shortens, and the resulting precipitates at the application of the creep load are refined (cf. Figure 2c and Figure 3). This leads to a slightly increased primary creep strain (Figure 6a), but prolonged time to reach the minimum creep rate, (i.e., time until the onset of tertiary creep, because of the absence of a classical secondary, steady-state creep stage) of approximately 2250 h (Figure 6b) and improved creep rupture time (6133 h). If a second precipitation treatment stage for 1 h at 650 °C (subsequent WQ) is added, the coverage of sub-grain boundaries and dislocations by Laves phase precipitates as well as intragranular precipitation increases (cf. Figure 2d), leading to a higher number of particles below 20 nm in size (cf. Figure 3). As a consequence, the material yields diminished primary creep strain (Figure 6a), shortened the time to minimum creep rate and the onset of tertiary creep (Figure 6b: 1750 h), but further improved creep rupture time (6597 h). In comparison to two-step annealing, single-step precipitation annealing at the envisaged creep temperature (1 h at 650 °C) causes increased primary creep strain, because of more pronounced recovery. Without the particle-refining effect of the low-temperature first stage, the particle size distribution “flattens” out towards larger particles (Figure 3), which are more stable against coarsening during creep. This causes the creep rupture time to rise to 7310 h. In the as-rolled material, increased dislocation density from the rolling process is a dominating factor for creep performance. Recovery of these excess dislocations during heat treatment is a creep performance-diminishing process, which competes with precipitation as a creep performance-enhancing process. Although heat treatment of rolled material compromises these conflicting issues, an approximately 40% improvement (from 5334 h to 7310 h) was achieved by precipitation annealing of the as-rolled steel.

#### 3.3.2. Recrystallized Material

Combining the findings from heat treatment of the as-rolled steel, the recrystallized variant was low temperature pre-annealed at 540 °C for 5 h in any case to compensate for (i) the delay in the onset of precipitation caused by recrystallization, (ii) for decreased PFZ width, and (iii) particle refinement in the subsequent 650 °C annealing stage.

**Figure 7:**
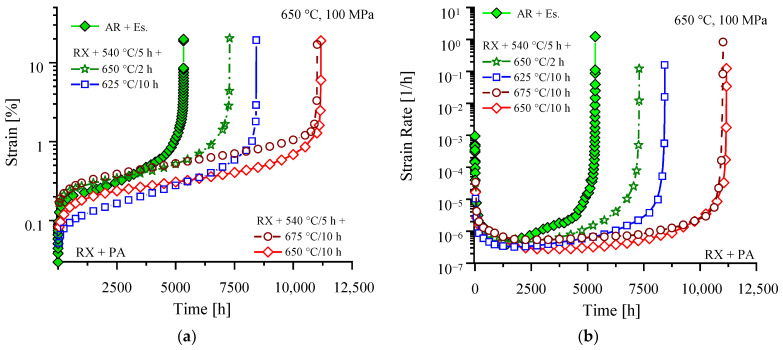
The 650 °C/100 MPa creep (**a**) and creep rate (**b**) curves of recrystallized material after various precipitation heat treatments.

After recrystallization and 540 °C pre-annealing, additional tempering at 650 °C for 2 h gives a moderate improvement in creep rupture time of about 35% (7294 h, Figure 7a). In comparison to the rolled variant, the extent of primary creep strain is slightly enlarged (because of low dislocation density), but the minimum creep rate (5.6 × 10^−7^ h^−1^) is reduced, and the onset of tertiary creep delayed (2250 h, Figure 7b). After prolonged annealing of 10 h, the particle size distribution (Figure 5a) tends to higher overall number of precipitates and extends towards the size fractions from 40 to 120 nm, which are lacking after only 2 h of annealing. Consequently, the lowest minimum creep rate (2.8×10^−7^ h^−1^), latest onset of tertiary creep (4250 h), and approximately doubled creep rupture time (11,171 h) is reached. Reduction in annealing temperature to 625 °C results in a much higher number of small precipitates (Figure 5b), which causes diminished primary creep strain (Figure 7a), but proves less stable during creep and for this reason leads to the earliest onset of tertiary creep (1750 h, Figure 7b) of the RX + PA variants. Nevertheless, a rise in creep rupture time to 8430 h was achieved. Precipitation treatment at 675 °C, on the other hand, induces reduced numbers of medium-size precipitates (20 to 100 nm, Figure 5b), but improves the number of particles in the size range from 100 to 160 nm, which in turn causes the highest extent of primary creep strain (Figure 7a). Obviously, due to the supreme stability of the particle size distribution, the rupture time reached (11,016 h, Figure 7a) is nevertheless almost comparable to annealing at 650 °C. The creep results are summarized in Table 4.

### 3.4. Creep Property Evaluation

**Figure 8:**
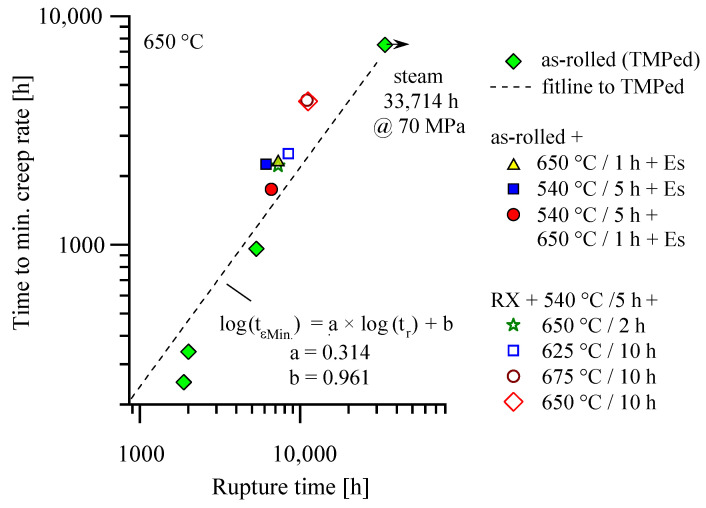
Time to minimum creep rate vs. time to rupture relations of the heat-treated materials in relation to the as-rolled variant (data and fitline of as-rolled, i.e., thermomechanically processed (TMPed), materials reproduced from [9]).

The time to minimum creep rate/time to rupture relation evaluated for the thermomechanically treated material variant

log(t_εMin._) = a × log(t_r_) + b (1)

with a = 0.314 and b = 0.961 [9] is displayed in Figure 8. Starting from both the as-rolled and recrystallized initial states, HiperFer steel acceptably obeys this relation in the precipitation heat-treated state, too. For this reason, the same regression constants apply, and the tertiary stage dominates with approximately 70% of creep life in case of the heat-treated materials, too.

Creep deformation of HiperFer steel for this reason is easy to monitor and can be handled with existing procedures.

## 4. Conclusions and Outlook

A suitable precipitation heat treatment window to make thermomechanical processing of HiperFer steel optional was evaluated. In recrystallized, low dislocation density HiperFer steel homogeneous dispersion and favorable particle size distribution of Laves phase precipitates within the alloy matrix is achievable in a temperature range from 625 to 675 °C and corresponding times from 10 (for all temperatures) down to 1 h (at 675 and 650 °C). The same applies to as-rolled (i.e., TMPed) material.

In both the TMPed and RX states, precipitation of strengthening Laves phase particles starts on grain boundaries [19,20,25], followed by intragranular precipitation, forming particle-free zones at grain boundaries, which must be controlled to reach favorable mechanical properties at high temperature. Pre-annealing at a comparably low temperature of 540 °C was effective in minimizing PFZ width and refinement of particles. In comparison to the TMPed state, precipitation was found to be delayed, intragranular precipitation more homogenous, and intergranular precipitation less pronounced in the recrystallized material. Pre-annealing at 540 °C further counterbalanced the delay in the onset of precipitation in the RXed state.

Precipitate population and creep response can be well correlated. HiperFer yields higher primary creep strain but diminished minimum creep rate and delayed time to the onset of tertiary creep in the recrystallized and precipitation annealed state. In comparison to the TMPed state, an approximate improvement in rupture time of 110% (TMPed: 5334 h vs. RXed + 540 °C/10 h/WQ + 650//675 °C/10 h/WQ: 11,171//11,016 h) was achieved.

However, the presented results may not represent the optimum considering the creep strength of HiperFer-type steel. Recently published research demonstrated improved creep rupture ductility [26] by alloying with small quantities of boron. Higher contents of W, Nb [27], and Si [28] directly impact the precipitation process. For these reasons, further-improved, technically viable, and well-monitorable creep strength potential should be accessible via combined alloying and tuned heat treatment and/or thermomechanical processing.

As a summary, it can be stated that heat treatment in the outlined parameter field provides improved creep strength both in the TMPed and RXed initial states. With the best creep rupture times being encountered in the recrystallized and precipitation annealed state, thermomechanical processing is not mandatory. With a thermomechanically treated variant for forgings and a recrystallized and precipitation annealed variant for pipes and tubes, the HiperFer alloying philosophy provides processing flexibility along with superior resistance to creep deformation and steam oxidation.

## Figures and Tables

**Table 1:** Chemical composition of the HiperFer trial steel (in wt.%).

Batch-ID:	C	N	Cr	Mn	Si	Nb	W
**HiperFer 17Cr2**	<0.01	<0.01	17.1	0.18	0.25	0.63	2.41

**Table 2:** Parameters applied in processing of the trial steel.

ID:		Soaking	Rolling		Interpass Annealing		Cooling:
	Temperature [°C]:	Time [Min.]:	Steps [-]:	Thickness Reduction [%]:	Steps [-]:	Time [Min.]:	
_4	1140	120	-	-	-	-	-
	950–920	-	4	65	-	-	--
	920	-	final	10	-	-	
	1085	-	-	-	3	10	-
	-	-	-	-	-	-	Water (stir)

**Table 3:** Parameters applied in recrystallization and precipitation heat treatment.

RX:	PA Stage 1	PA Stage 2	“Equil. Stage”
-	-	-	650 °C/1 h/W
		650 °C/1 h/W	
	540 °C/5 h/WQ	650 °C/1 h/W	
		-	
1075 °C/15 min/WQ		650 °C/1 h/W	
		650 °C/2 h/W	
		650 °C/10 h/W	
		625 °C/10 h/W	
		675 °C/10 h/W	

**Table 4:** Summary of creep testing results at heat-treated HiperFer steel (650 °C/100 MPa).

State:	PA Stage 1	PA Stage 2	“ES”	t_r_ [h]:	ε_r_ [%]:
AR	-	-	650 °C/1 h/W	5334	19.8
		650 °C/1 h/W		7038	12.1
	540 °C/5 h/WQ			6596	9.0
		-		6132	12.7
RX		650 °C/1 h/W		7294	20.3
		650 °C/2 h/W		7278	16.6
		650 °C/10 h/W		11,171	18.9
		625 °C/10 h/W		8430	19.3
		675 °C/10 h/W		11,016	17.0

## Data Availability

Not applicable.
